# Postoperative Atrial Fibrillation After Coronary Artery Bypass Grafting—Clinical, Demographic, and Intraoperative Predictors: A Multicenter Observational Study

**DOI:** 10.3390/healthcare14050690

**Published:** 2026-03-09

**Authors:** Kyriakos Alexandrou, Nicos Middleton, Maria Kyranou, Pavlos Sarafis

**Affiliations:** 1 Department of Nursing, Cyprus University of Technology, Limassol 3036, Cyprus; kya.alexandrou@edu.cut.ac.cy (K.A.); nicos.middleton@cut.ac.cy (N.M.); maria.kyranou@cut.ac.cy (M.K.); 2 Department of Nursing, University of Thessaly, 41500 Larisa, Greece

**Keywords:** coronary artery bypass grafting, postoperative arrhythmias, atrial fibrillation, predictors, cardiac surgery

## Abstract

**Background:** Postoperative arrhythmias, especially atrial fibrillation (AF), are common complications of coronary artery bypass grafting (CABG) associated with prolonged hospitalization and adverse outcomes. This study aimed to assess the incidence of postoperative AF and identify demographic, clinical, and intraoperative predictors in CABG patients in Cyprus. **Methods:** This prospective, multicenter observational study was conducted in three cardiac surgery centers in Cyprus between September 2022 and April 2023. Adult elective CABG patients in preoperative sinus rhythm were included; emergency cases and those with prior arrhythmias or conduction disturbances were excluded. Data on demographic, clinical, intraoperative, and postoperative variables, including norepinephrine infusion duration, were collected daily. Postoperative arrhythmias were systematically recorded during hospitalization. Statistical analyses included descriptive statistics, bivariate tests, and multivariable logistic regression to identify independent predictors of postoperative atrial fibrillation. **Results:** Among 102 patients (mean age 66.8 years, 78.4% male), postoperative arrhythmias occurred in 26.5%. AF was most frequent (20.6%), followed by ventricular tachycardia (2.9%), atrial tachycardia (1.0%), atrioventricular block (1.0%), and one fatal asystole. Key independent predictors of AF were increasing age, atrial enlargement, severely reduced left ventricular ejection fraction (<30%), and prolonged norepinephrine infusion. **Conclusions:** Postoperative AF remains a prevalent and clinically significant complication after CABG. The association with norepinephrine duration underscores the importance of careful hemodynamic management. Further studies and AI-based prediction models may enhance individualized prevention strategies.

## 1. Introduction

Coronary artery bypass grafting (CABG) is an established surgical treatment for advanced coronary artery disease, with over 400,000 procedures performed annually in the United States alone [1,2]. Despite its proven long-term benefits compared to percutaneous coronary interventions, CABG remains a complex procedure associated with significant postoperative complications [3]. Postoperative atrial fibrillation (POAF) is linked to increased risks of stroke, thromboembolism, cardiac arrest, and mortality, and is often associated with longer hospital stays and substantially higher medical costs [4].

Postoperative arrhythmias, particularly atrial fibrillation (AF), are among the most frequent and clinically consequential complications, affecting 15–40% of CABG patients [5,6]. While many arrhythmias are transient, persistent AF is linked to prolonged hospitalization, increased morbidity, stroke risk, and mortality, causing a notable burden on healthcare systems worldwide [7,8]. Established risk factors include older age, male sex, atrial enlargement, reduced left ventricular ejection fraction (LVEF), chronic obstructive pulmonary disease, and concomitant valve surgery [9].

Other arrhythmias such as ventricular tachycardia or atrioventricular blocks, though less common, also contribute to adverse outcomes after CABG [6]. Despite growing clinical awareness, there is still limited understanding of how specific intraoperative factors and early postoperative management contribute to the development of POAF [3]. Given the multifactorial etiology of postoperative arrhythmias, identifying demographic, clinical, and intraoperative predictors is crucial to improve prevention and management strategies [5].

In several healthcare systems, efforts have been made to implement early recognition protocols and targeted prophylaxis, though approaches remain inconsistent and are not yet standardized [10]. At the same time, future-oriented strategies such as real-time risk monitoring and the use of artificial intelligence in perioperative care are being explored but remain in early stages of implementation [7].

Within this broader context, the Cypriot setting provides an opportunity to examine these predictors across public and private hospitals under a unified healthcare framework. This multicenter observational study aimed to assess the incidence of postoperative AF after CABG and to identify its clinical, demographic, and intraoperative predictors in a representative real-world population, with relevance for broader clinical applications.

## 2. Methods

### 2.1. Study Design and Setting

This study was designed as a prospective, multicenter observational cohort study aimed at evaluating the incidence and determinants of postoperative arrhythmias in patients undergoing CABG across Cyprus. Data were collected between September 2022 and April 2023.

Recruitment took place at three cardiac surgery centers, two private institutions and one public hospital that all operate within the General Healthcare System (GHS) and collectively represent the majority of CABG-performing facilities nationwide. The inclusion of three out of four eligible hospitals, situated in the major urban centers of Nicosia and Limassol, enhanced the representativeness of the sample and reduced the risk of selection bias. Nicosia hospitals mainly serve patients from the districts of Nicosia, Larnaca, and Famagusta, whereas the Limassol center served primarily the Limassol and Paphos districts. The one non-participating hospital, also a private institution in Nicosia, serves a patient population demographically similar to that of a participating center, mitigating concerns regarding potential systematic differences.

### 2.2. Study Population

Eligible participants were adult patients (≥18 years) scheduled for elective CABG, with or without concomitant valve surgery, who provided written informed consent prior to enrolment. Only patients in sinus rhythm during the preoperative evaluation were included to allow assessment of new-onset postoperative arrhythmias.

Exclusion criteria comprised emergency CABG cases, patients unable to provide consent due to sedation or cognitive impairment, and those with a history of permanent arrhythmias or advanced conduction disturbances before surgery.

All patients were transferred immediately post-surgery to the intensive care unit for standardized monitoring, followed by ward-based care. Although each hospital employed its own postoperative management protocols, the core principles of monitoring and care were consistent across centers, supporting comparability of outcomes.

### 2.3. Study Instruments

Data collection was performed using a structured clinical data form specifically developed for this study to systematically capture comprehensive perioperative information in patients undergoing CABG (see Supplementary File S1).

Clinical history focused on cardiovascular and non-cardiovascular comorbidities associated with AF risk, including renal failure, hypertension, hypercholesterolemia, asthma, heart failure, chronic respiratory disease, and prior arrhythmias, aligning with the comorbidity patterns observed in the study cohort. The form also included sociodemographic variables such as age, sex, marital status, educational attainment, and occupational status, ensuring detailed patient profiling consistent with the demographics reported.

Preoperative cardiac assessments documented key structural heart variables, notably LVEF, which was categorized into three groups: less than 30%, between 30% and 50%, and greater than 50%. These values were derived from preoperative transthoracic echocardiography using standardized measurement protocols. Atrial enlargement was assessed preoperatively using transthoracic echocardiography. Left atrial size was quantified according to ASE/EACVI guidelines using the left atrial volume index (LAVI), with atrial enlargement defined as LAVI > 34 mL/m^2^ and measurements indexed to body surface area. Procedural data recorded included concomitant valve surgery, number of grafts, and use of cardiopulmonary bypass, consistent with operative details shared in the results [11].

Postoperative monitoring emphasized detailed hemodynamic support data, specifically the precise duration of norepinephrine infusion as a vasoactive drug, alongside length of hospital stay. Arrhythmic events were systematically recorded, classified by type and timing during hospitalization across all participating centers, in line with the postoperative arrhythmia data presented.

Data collection was performed prospectively by trained members of the research team at each participating hospital, including cardiac surgery nurses and clinical researchers. Data were recorded daily during the patients’ hospitalization, using a standardized data collection form. All information was obtained through direct chart review and cross-verified with patient medical records and nursing documentation to ensure completeness and accuracy. The data collection form was piloted prior to study initiation to enhance clarity and feasibility, and minor adjustments were made to standardize recording procedures across sites.

### 2.4. Statistical Analysis

Descriptive statistics were reported as means ± standard deviation or medians with interquartile ranges for continuous variables, and frequencies (percentages) for categorical variables. The normality of continuous variables was assessed with the Shapiro–Wilk test; as most distributions deviated from normality, non-parametric methods were used throughout the analysis. The incidence of postoperative atrial fibrillation (AF) was calculated as a proportion of the entire study cohort.

Bivariate comparisons between patients with and without postoperative AF were performed using the Mann–Whitney U test for continuous variables, and χ^2^ or Fisher’s exact test for categorical variables depending on expected cell counts. For variables with more than two categories, the Kruskal–Wallis test was applied, with Bonferroni-adjusted post hoc pairwise comparisons where appropriate.

For categorical variables such as left ventricular ejection fraction (LVEF), conventional groupings were used for descriptive and bivariate analyses (<40%, 41–50%, >50%). To enhance statistical power and interpretability in the multivariable regression, LVEF was dichotomized as <30% versus ≥30%, consistent with published clinical and methodological guidance.

Multivariable logistic regression was carried out to identify independent predictors of postoperative AF. The model included age, sex, history of renal failure, hypertension, hypercholesterolemia, asthma, prior arrhythmia, heart failure, chronic respiratory disease, atrial enlargement, LVEF (<30% vs. ≥30%), valve surgery, number of grafts, duration of norepinephrine infusion, and days of hospital stay. The “hospital site” variable was initially considered but removed from the final regression model following exploratory analysis, due to lack of statistical significance, absence of theoretical association with arrhythmia development, and to avoid issues stemming from improper coding as a continuous variable.

Odds ratios (ORs) and their 95% confidence intervals (CIs) were calculated for all predictors, with statistical significance set at a two-tailed *p*-value < 0.05. Statistical analyses were performed using IBM SPSS Statistics version 29.0.

Sociodemographic variables such as education, residence, marital status, and employment status were collected for descriptive purposes but were not included in the regression analysis, as they are not established predictors of postoperative AF and showed no meaningful associations in exploratory analyses.

### 2.5. Ethical Considerations

This study adhered to the ethical principles outlined in the Declaration of Helsinki and received formal approval from the Cyprus National Bioethics Committee (approval number: 2021.01.153) as well as from the State Health Services Organization (for access to the public hospital). Additionally, administrative permissions were obtained from the management of all three participating hospitals prior to the commencement of data collection.

Eligible patients were approached during their preoperative assessment by members of the research team, who provided comprehensive oral and written information regarding the study’s objectives, procedures, and potential risks and benefits. Emphasis was placed on the voluntary nature of participation, confidentiality of data, and the assurance that refusal or withdrawal would have no impact on their clinical care.

Written informed consent was obtained from all participants prior to surgery and before any study-related data collection. Patients were afforded sufficient time to consider their participation and to seek clarification on any questions or concerns.

There is no clinical trial number.

## 3. Results

### 3.1. Demographics and Baseline Characteristics

The demographic characteristics of the study population are summarized in Table 1.

The study cohort consisted of 102 patients undergoing CABG, most of whom were male (78.4%), with a mean age of 66.8 years: the majority fell within the 60–79-year range. Patients originated from all major districts of Cyprus, with distributions reflecting regional population sizes. Almost half of the procedures were performed in the public tertiary hospital, while the remainder were divided between two private centers. Educational attainment ranged from primary to tertiary levels, and marital status was predominantly married, consistent with the age profile of the cohort. Employment data showed that slightly over half of the participants were retired or not working. These variables were collected to characterize the study population comprehensively but were not used in the inferential analyses.

### 3.2. Postoperative Arrhythmias

Among the 102 patients who entered surgery in sinus rhythm, 27 (26.5%) developed at least one arrhythmic event during hospitalization, while the remaining 75 (73.5%) maintained sinus rhythm throughout the postoperative period.

**Figure 1:**
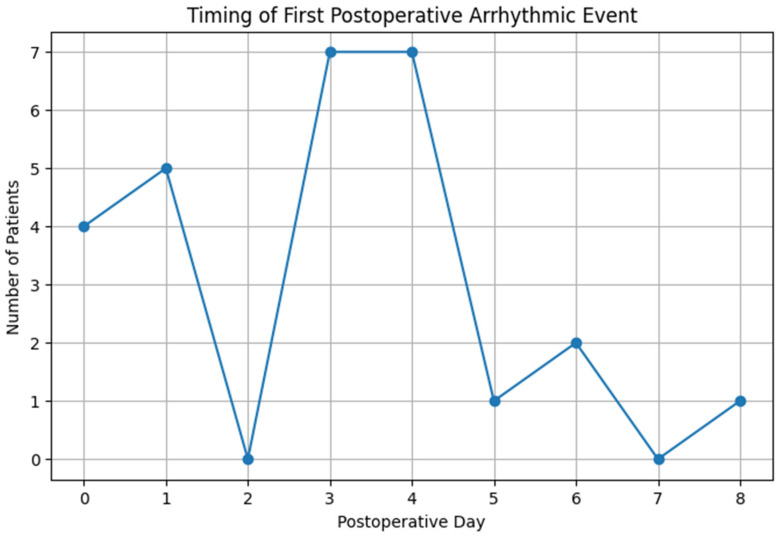
The timing of the first postoperative arrhythmic events during hospitalization.

The most common arrhythmia was AF, observed in 21 patients (20.6%). Sustained ventricular tachycardia with pulse occurred in 3 patients (2.9%), whereas one patient developed atrial tachycardia and another experienced second-degree atrioventricular block (Mobitz type II). These represent all arrhythmic events observed in the cohort. There was one postoperative death in the cohort, resulting from cardiac arrest due to asystole. The single postoperative death due to asystole is included in Figure 1 as a first recorded event but is not classified as an arrhythmia in the results, in accordance with clinical definitions. The timing of first postoperative arrhythmic events during hospitalization is illustrated in Figure 1.

The majority of arrhythmic events occurred between the third and fourth postoperative days (*n* = 7 each, 25.9%). Smaller peaks were noted on the first postoperative day (*n* = 5, 18.5%) and on the day of surgery (*n* = 4, 14.8%), whereas isolated cases occurred on the fifth (*n* = 1), sixth (*n* = 2), and eighth (*n* = 1) postoperative days.

### 3.3. Postoperative Atrial Fibrillation by Demographic and Clinical Characteristics

Table 2 presents the bivariate associations between selected demographic and clinical characteristics and the occurrence of postoperative atrial fibrillation (AF). No statistically significant associations were identified for any of the examined variables (all *p* > 0.05). No variables were significantly associated with POAF in the bivariate analyses; however, multivariable logistic regression identified several independent predictors after adjustment for confounding.

The results of the multivariable logistic regression are summarized in Table 3.

Specifically, postoperative AF was observed in 21 patients (20.6%), with a slightly higher proportion among females (27.3%) compared to males (18.8%), although this difference was not significant (*p* = 0.384). Age distribution was similar across AF and non-AF groups, with no apparent trend or significant association (*p* = 0.914).

The incidence of AF did not differ significantly by hospital site (*p* = 0.175), although a higher proportion of cases was noted in one of the private centers (42.9%). Clinical comorbidities such as heart failure (*p* = 0.291), renal failure (*p* = 0.132), and atrial enlargement (*p* = 0.262) showed modest differences in proportions between groups but did not reach statistical significance.

Regarding left ventricular ejection fraction (LVEF), patients with AF were slightly more represented in the <40% and >50% categories; however, no significant association was detected (*p* = 0.522). Similarly, the presence of valve surgery (*p* = 0.682), number of bypass grafts (*p* = 0.699), and duration of norepinephrine support (*p* = 0.951) were not significantly related to AF occurrence.

Finally, length of hospital stay, grouped into three categories, was comparable between groups (*p* = 0.836), indicating no clear relationship with postoperative arrhythmia onset. The detailed distribution of demographic and clinical variables according to postoperative atrial fibrillation status is shown in Table 2.

The results of the multivariable logistic regression are summarized in Table 3 and presented in detail below. Increasing age was significantly associated with the development of postoperative AF after CABG, with each additional year conferring a 16% increase in the odds of arrhythmia (OR = 1.16, 95% CI: 1.01–1.33, *p* = 0.04). This finding demonstrates the incremental risk posed by advancing age and is consistent with the recognized contribution of electrical and structural atrial remodeling in older patients.

Atrial enlargement was also identified as an independent predictor, increasing the likelihood of AF by approximately 14% (OR = 1.14, 95% CI: 1.09–6.67, *p* = 0.03). The strong association between atrial enlargement and arrhythmic outcomes underscores the role of underlying structural changes that may serve as arrhythmogenic substrates following surgery.

Reduced LVEF (<30%) was among the most powerful determinants of AF, more than tripling the odds of postoperative arrhythmia (OR = 3.27, 95% CI: 1.43–7.48, *p* = 0.01). This highlights the critical influence of impaired cardiac function, where increased filling pressures and neurohormonal activation may predispose to atrial irritability and fibrillation.

Τhe duration of norepinephrine infusion was strongly correlated with AF occurrence, with each additional day of vasoactive support raising the risk by 37% (OR = 1.37, 95% CI: 1.14–1.64, *p* = 0.001). This result highlights the significant impact of sustained adrenergic stimulation and perioperative hemodynamic stress on postoperative rhythm stability.

By contrast, variables such as sex (OR = 1.80, *p* = 0.57), history of heart failure (OR = 0.12, *p* = 0.09), renal failure (OR = 0.11, *p* = 0.13), valve surgery (OR = 0.04, *p* = 0.12), number of grafts (OR = 1.47, *p* = 0.53), and length of hospital stay (OR = 1.11, *p* = 0.58) did not reach statistical significance in this cohort, although some demonstrated trends that may warrant further investigation in larger populations.

## 4. Discussion

In this multicenter cohort of patients undergoing CABG in Cyprus, postoperative AF emerged as the most frequent arrhythmic complication, in line with rates reported internationally [12,13,14]. While descriptive and bivariate analyses did not reveal significant associations with individual demographic or clinical variables, the multivariable model identified advanced age, atrial enlargement, markedly reduced left ventricular ejection fraction, and prolonged norepinephrine support as independent predictors. These findings highlight the multifactorial nature of AF development after cardiac surgery and the value of adjusted analytic approaches in uncovering associations that are not evident in unadjusted comparisons. Importantly, all affected patients were promptly treated and discharged in sinus rhythm, a favorable outcome associated with reduced complications and improved long-term prognosis [5,15].

Multivariate analysis revealed four independent predictors of postoperative AF: advanced age, atrial enlargement, reduced left ventricular ejection fraction (LVEF < 30%), and prolonged norepinephrine infusion. The association between aging and AF is well established, likely due to age-related structural and electrical remodeling of atrial tissue, leading to conduction heterogeneity and increased arrhythmogenic potential [16,17]. Similarly, atrial enlargement constitutes a known structural substrate for AF, particularly in the context of surgical stress and inflammation. Reduced LVEF reflects impaired cardiac function, which increases the susceptibility to AF through neurohormonal and hemodynamic alterations. Moreover, prolonged norepinephrine support may contribute to sustained adrenergic stimulation, exacerbating postoperative arrhythmogenesis [4].

Reduced LVEF emerged as a significant risk factor, reflecting the role of impaired myocardial function in promoting postoperative atrial electrical instability. Notably, a strong association was observed between prolonged norepinephrine infusion and POAF. This may be mechanistically explained by enhanced sympathetic activity, as norepinephrine binds to β1-adrenergic receptors via pathways originating from cervical and upper thoracic sympathetic ganglia. The resulting increase in adrenergic drive accompanied by reduced vagal tone can shorten atrial refractory periods, increase myocardial excitability and preload, thereby creating a pro-arrhythmic substrate [4,18,19]. In this context, continuous hemodynamic monitoring and the timely titration of vasopressors by nursing staff, with the goal of early weaning and discontinuation where clinically feasible, represent critical steps in mitigating this modifiable risk factor [20]. These findings, identified through multivariable logistic regression analysis that controls for confounding, highlight the importance of targeted perioperative management to reduce POAF incidence beyond what descriptive or bivariate analyses alone might reveal.

It is important to emphasize, based on the results presented in Table 2 and Table 3, that the bivariate analysis did not reveal statistically significant associations for these variables, underscoring the necessity of comprehensive multivariable modeling to uncover independent predictors. This difference in analytical approach and consequent findings constitutes a major methodological strength of the study.

Our findings are largely consistent with previous studies examining predictors of POAF following CABG. For example, Seo et al. (2021) conducted a systematic review and meta-analysis of prospective studies investigating perioperative risk factors for new-onset POAF after CABG. Their analysis identified several significant predictors, including advanced age, reduced left ventricular ejection fraction, hypertension, renal dysfunction, and elevated preoperative creatinine levels. Among these, our study similarly confirmed the importance of advanced age and impaired ventricular function as key contributors to POAF development. However, some comorbid conditions identified in previous studies, such as hypertension and renal dysfunction, did not reach statistical significance in our cohort, which may reflect differences in patient characteristics or the relatively smaller sample size of our population [16]. Similarly, the meta-analysis by Kerwin et al. (2020), which synthesized data from over 17,000 patients, emphasized age and atrial size as robust risk factors for POAF. While LVEF was included in most adjusted models of that analysis, it was not consistently reported as an independent predictor contrary to our findings, where severely reduced LVEF (<30%) emerged as one of the strongest determinants. This divergence may reflect the narrower LVEF thresholds used in our cohort or a more pronounced role of systolic dysfunction in this specific patient population [21].

Of particular note, the association between prolonged norepinephrine infusion and POAF a key finding in our study has not been extensively evaluated in prior large-scale analyses. However, a systematic review by Seo et al. (2021) identified postoperative inotrope use as a significant risk factor for the development of POAF after CABG. Our findings extend this evidence by providing quantitative data demonstrating that each additional day of norepinephrine administration was associated with a 37% increase in AF risk, suggesting that sustained catecholaminergic stimulation may represent an underrecognized modifiable factor in postoperative arrhythmogenesis [16].

It is also worth noting that although renal failure and heart failure showed trends toward increased risk in our study, they did not reach statistical significance. In contrast, a systematic review by Seo et al. (2021) identified renal failure as a significant risk factor associated with the development of postoperative atrial fibrillation after CABG. This discrepancy may relate to differences in baseline renal function, sample size, or perioperative management strategies [16]. It should also be noted that all surgeries in this cohort were performed using conventional on-pump CABG, with no off-pump cases recorded; therefore, potential differences in AF incidence between on-pump and off-pump techniques could not be assessed in this study.

Collectively, our results support several well-established predictors of POAF while also highlighting potentially novel clinical factors, such as the duration of vasoactive support, that warrant further investigation. Incorporating these variables into future risk stratification models could enhance individualized perioperative monitoring and prophylaxis.

Future studies should focus on validating key predictors of POAF in larger, multicenter cohorts with longer follow-up to assess both incidence and long-term recurrence. Inclusion of more heterogeneous populations across healthcare systems would also improve the external applicability of risk models [21].

Looking ahead, the integration of artificial intelligence (AI) into perioperative risk assessment may provide new opportunities for predicting and preventing POAF. Emerging machine learning and deep learning models have shown high accuracy in detecting atrial fibrillation and forecasting arrhythmic risk based on ECG and clinical parameters [22,23]. More recent transformer-based systems capable of processing multimodal inputs—including echocardiography, hemodynamics, and clinical notes—may enable real-time risk identification [24,25]. Such tools could support clinicians by signalling elevated risk profiles and guiding timely interventions, including vasopressor adjustment or targeted prophylaxis [26,27,28]. Although AI was not used in the present study, these technologies represent a promising future direction for personalized perioperative management.

### Limitations

This study should be interpreted in light of several limitations. First, the relatively small cohort size, particularly within subgroups such as female patients and those with severely reduced ventricular function, may have limited the statistical power to detect additional independent predictors of arrhythmia. Second, the observational design precludes causal inference, and unmeasured confounding factors—such as variations in perioperative clinical management protocols, genetic predispositions, and socioeconomic determinants—may have influenced the observed associations. Third, although data were collected prospectively across multiple hospitals, differences in perioperative practices and management strategies may have introduced variability that was not fully accounted for.

Furthermore, the lack of standardized documentation of key surgical strategy elements (including myocardial protection technique, bypass method, cardiopulmonary bypass duration, and aortic cross-clamp management) prevented their incorporation into the multivariable analysis. These variables were also outside the predefined aims of the study and therefore were not included in the structured data collection framework.

Additionally, arrhythmic events were recorded only during the index hospitalization, based on routine in-hospital monitoring; therefore, transient or asymptomatic episodes that may have occurred after discharge could not be identified. Finally, the focus on short-term postoperative outcomes limits the ability to assess long-term AF recurrence, symptom burden, or the broader clinical implications following discharge.

## 5. Conclusions

Postoperative atrial fibrillation remains a frequent and clinically significant complication following CABG, with an observed incidence of 20.6% in this multicenter Cypriot cohort. The identification of increasing age, atrial enlargement, reduced left ventricular ejection fraction, and prolonged norepinephrine infusion as independent predictors underscores the multifactorial nature of arrhythmogenesis in this context. These findings highlight the importance of comprehensive preoperative assessment and judicious postoperative management to mitigate risk. The potential role of real-time artificial intelligence systems in enhancing arrhythmia prediction and prevention offers a promising avenue for future research and clinical innovation. Ultimately, targeted strategies based on individualized risk profiles may improve patient outcomes and reduce the burden of postoperative arrhythmias in cardiac surgical populations. Future studies should focus on validating these predictors in larger, diverse cohorts and exploring the integration of AI tools for personalized perioperative care.

## Figures and Tables

**Table 1:** Baseline characteristics of the study population.

Variable	*n* (%)
**Sex**	
Male	80 (78.4%)
Female	22 (21.6%)
**Age**	
<50	6 (5.9%)
50–59	12 (11.8%)
60–69	39 (38.2%)
70–79	41 (40.2%)
≥80	4 (3.9%)
**Education**	
Primary school	24 (23.5%)
Secondary school	20 (19.6%)
High school/technical	36 (35.3%)
Bachelor’s degree	18 (17.6%)
Master’s degree	3 (2.9%)
Doctorate	1 (1.0%)
**Residence**	
Nicosia	38 (37.3%)
Limassol	29 (28.4%)
Larnaca	20 (19.6%)
Paphos	8 (7.8%)
Famagusta	7 (6.9%)
**Hospital**	
Private 1	25 (24.5%)
Private 2	28 (27.5%)
Public	49 (48.0%)
**Marital status**	
Married	88 (86.3%)
Divorced	6 (5.9%)
Widowed	8 (7.8%)
**Employment**	
Yes	45 (44.1%)
No	57 (55.9%)

**Table 2:** Demographic and baseline clinical characteristics of patients with and without postoperative atrial fibrillation.

Variable	No AF (*n* = 81)	AF (*n* = 21)	*p*-Value
**Sex**			
Male	65 (80.2%)	15 (71.4%)	0.384
Female	16 (19.8%)	6 (28.6%)	
**Age group**			
<50	4 (4.9%)	2 (9.5%)	0.914
50–59	10 (12.3%)	2 (9.5%)	
60–69	32 (39.5%)	7 (33.3%)	
70–79	32 (39.5%)	9 (42.9%)	
≥80	3 (3.7%)	1 (4.8%)	
**Hospital**			
Private 1	20 (24.7%)	5 (23.8%)	0.175
Private 2	19 (23.5%)	9 (42.9%)	
Public	42 (51.9%)	7 (33.3%)	
**Heart Failure**			
Yes	18 (22.2%)	7 (33.3%)	0.291
No	63 (77.8%)	14 (66.7%)	
**Renal Failure**			
Yes	9 (11.1%)	5 (23.8%)	0.132
No	72 (88.9%)	16 (76.2%)	
**Atrial** **Enlargement**			
Yes	21 (25.9%)	3 (14.3%)	0.262
No	60 (74.1%)	18 (85.7%)	
**LVEF Category**			
<40%	14 (17.3%)	5 (23.8%)	0.522
41–50%	38 (46.9%)	7 (33.3%)	
>50%	29 (35.8%)	9 (42.9%)	
**Valve Surgery**			0.682
No	54 (66.7%)	13 (61.9%)	
Yes	27 (33.3%)	8 (38.1%)	
**Number of Grafts**			0.699
1	15 (18.5%)	3 (14.3%)	
2	26 (32.1%)	8 (38.1%)	
3	25 (30.9%)	8 (38.1%)	
4	15 (18.5%)	2 (9.5%)	
**Norepinephrine Duration**			0.951
≤2 days	69 (85.2%)	18 (85.7%)	
≥3 days	12 (14.8%)	3 (14.3%)	
**Hospital Stay (days**)			0.836
≤5 days	7 (8.6%)	1 (4.8%)	
6–10 days	64 (79.0%)	17 (81.0%)	
≥11 days	10 (12.3%)	3 (14.2%)	

*p*-values calculated using Pearson’s chi-square or Mann–Whitney U test as appropriate.

**Table 3:** Intraoperative characteristics and perioperative factors associated with postoperative atrial fibrillation.

Variable	Odds Ratio	95% CI Lower	95% CI Upper	*p*-Value
Age	1.16	1.01	1.33	0.04
Gender (Male vs. Female)	1.80	0.24	13.59	0.57
Heart Failure	0.12	0.01	1.39	0.09
Renal Failure	0.11	0.01	1.84	0.13
Atrial Enlargement	2.70	1.09	6.67	0.03
LVEF	3.27	1.43	7.48	0.01
Valve Surgery	0.04	0.00	2.43	0.12
Number of Grafts	1.47	0.44	4.83	0.53
Norepinephrine duration	1.37	1.14	1.64	0.001
Days of Hospital Stay	1.11	0.77	1.59	0.58

*p*-values calculated using Pearson’s chi-square or Mann–Whitney U test as appropriate.

## Data Availability

The datasets generated and analyzed during the current study are not publicly available due to patient privacy and ethical restrictions but are available from the corresponding author upon reasonable request.

## Supplementary Materials

The following supporting information can be downloaded at: https://www.mdpi.com/article/10.3390/healthcare14050690/s1, File S1: Clinical data collection form used for the collection of demographic, clinical, intraoperative, and postoperative data in patients undergoing coronary artery bypass grafting (CABG).
