# Supplementary material for: Postoperative Atrial Fibrillation After Coronary Artery Bypass Grafting—Clinical, Demographic, and Intraoperative Predictors: A Multicenter Observational Study
Source: Healthcare (Basel). 2026 Mar 9;14(5):690. doi: 10.3390/healthcare14050690 (PMC12985182; doi:10.3390/healthcare14050690)
Supplement: Supplementary file 1 [file healthcare-14-00690-s001.zip › healthcare-4140355-supplementary.pdf]

Research Study entitled:

“Quality of Life in Patients Undergoing Coronary Artery Bypass Grafting, Arrhythmias, and Medication Adherence.”

Please select the response that best represents you for each question by marking the appropriate option. In some cases, additional written clarification is required.

Demographic Information

1. **Sex:**
   Male
   Female
2. **Age:** ______________________
3. **Place of Residence:** ______________________
4. **Marital Status:**
   Married
   Divorced
   Single
   Widowed
   Partnered
5. **Education Level:**
   Primary School Graduate
   High School Graduate
   University Graduate
   Master’s Degree
   Doctoral Degree
6. **Are you currently employed?**
   Yes / No
7. **Are you retired?**
   Yes / No
8. **Family history:**
   Do you have a first-degree relative who has experienced an arrhythmia?

Medical History

Please select the response that best represents you for each question. In some cases, additional written clarification is required.

1. **Have you been diagnosed with any of the following conditions?**
   Please indicate all that apply:

Coronary artery disease
Hypertension
Heart failure
Diabetes mellitus
Hypercholesterolemia
Asthma
Renal failure
Chronic respiratory disease
Other (please specify): ______________________

2. **Have you undergone previous cardiac surgery?**
   Yes / No

3. **If yes, please specify the type of surgery and the year performed:**

---

4. **Have you experienced arrhythmia in the past?**
   Yes / No

Preoperative Period

1. **Left Ventricular Ejection Fraction (LVEF): ______** %

2. **Atrial or Ventricular dilation or hypertrophy (please specify):**

---

3. **Previous Myocardial Infarction:**
   Yes / No

4. **If yes, when did it occur and how many times?**

Intraoperative Period

1. **Type of procedure performed (please indicate):**
   Coronary Artery Bypass Grafting (CABG)
   CABG with Aortic Valve Repair
   CABG with Aortic Valve Replacement
   CABG with Mitral Valve Repair
   CABG with Mitral Valve Replacement
   Other procedure (please specify): ______________________

2. **Number of grafted vessels:**
   1 / 2 / 3 / 4
3. **Use of cardiopulmonary bypass (on-pump):**
   Yes / No
4. **Did arrhythmia occur intraoperatively?**
   Yes / No
5. **If yes, specify the type of arrhythmia:**

Postoperative Period

1. **Day of vasopressor (noradrenaline) discontinuation:**
   Day of surgery
   Postoperative Day 1
   Postoperative Day 2
   Postoperative Day 3
   Postoperative Day 4
   Postoperative Day 5
   Postoperative Day 6
   Other postoperative day (please specify): ___________
2. **Were intravenous inotropic agents (e.g., dobutamine) administered?**
   Yes / No
3. **If yes, for how many days were they administered?**

---

4. **Occurrence of arrhythmia postoperatively:**
   Yes / No
5. **Type and duration of arrhythmia:**

---

6. **Day of arrhythmia onset:**

---

7. **Was treatment administered for arrhythmia?**
   If yes, specify the pharmacological therapy:

---

8. **Length of stay in the Intensive Care Unit (days):**

---

9. **Acute kidney injury requiring dialysis:**
   Yes / No

10. **Readmission to the ICU before hospital discharge:**
    Yes / No

11. **Use of intra-aortic balloon pump:**
    Yes / No

12. **Total length of hospital stay (days):**

Biochemical Monitoring

13. **Please record serum potassium and magnesium levels during postoperative biochemical testing (Postoperative Days 1–20).**

14. **In the presence of arrhythmia in a patient with an arterial line, please record the potassium value in arterial blood gas analysis before and after the arrhythmia episode:**

Potassium level before arrhythmia: ___________
Potassium level after arrhythmia: ___________

15. **Type of arrhythmia:**
